# Short-Term and Two-Year Rate of Recurrent Cerebrovascular Events in Patients with Acute Cerebral Ischemia of Undetermined Aetiology, with and without a Patent Foramen Ovale

**DOI:** 10.5402/2011/959483

**Published:** 2011-12-15

**Authors:** Silvia Di Legge, Fabrizio Sallustio, Emiliano De Marchis, Costanza Rossi, Giacomo Koch, Marina Diomedi, Mauro Borzi, Francesco Romeo, Paolo Stanzione

**Affiliations:** ^1^Department of Clinical Neurological Sciences, Stroke Unit, University of Rome “Tor Vergata”, Viale Oxford 81, 00133 Rome, Italy; ^2^I.R.C.C.S. Santa Lucia Foundation, Via Ardeatina 306, 00179 Rome, Italy; ^3^Dipartimento di Cardiologia, Policlinico Tor Vergata, Università Tor Vergata, Viale Oxford 81, 00133 Rome, Italy

## Abstract

*Purpose*. We investigated stroke recurrence in patients with acute ischemic stroke of undetermined aetiology, with or without a patent foramen ovale (PFO). *Methods*. Consecutive stroke patients underwent to Transcranial Doppler and Transesophageal Echocardiography for PFO detection. Secondary stroke prevention was based on current guidelines. *Results*. PFO was detected in 57/129 (44%) patients. The rate of recurrent stroke did not significantly differ between patients with and without a PFO: 0.0% versus 1.4% (1 week), 1.7% versus 2.7% (1 month), and 3.5% versus 4.2% (3 months), respectively. The 2-year rates were 10.4% (5/48) in medically treated PFO and 8.3% (6/72) in PFO-negative patients (*P* = 0.65), with a relative risk of 1.25. No recurrent events occurred in 9 patients treated with percutaneous closure of PFO. *Conclusion*. PFO was not associated with increased rate of recurrent stroke. Age-related factors associated with stroke recurrence in cryptogenic stroke should be taken into account when patients older than 55 years are included in PFO studies.

## 1. Introduction

Controversy exists around the topic of PFO detection, its association with cerebral ischemia, and secondary prevention strategies [1]. Treatment options in patients with PFO include antiplatelet agents, anticoagulants, surgical closure, or percutaneous closure devices. A meta-analysis of the French PFO/atrial septal aneurysm (ASA) and PICCS (Patent Foramen Ovale in Cryptogenic Stroke Study) indicates that the risk of recurrent stroke or death after a cryptogenic stroke is not different for patients with a PFO compared to patients without a PFO when treated with either aspirin or warfarin, although aspirin is preferable (Class IIa, Level of Evidence B) [2, 3]. Warfarin is reasonable for high-risk patients who have other indications for oral anticoagulation such as those with an underlying hypercoagulable state or evidence of lower extremities/pelvic deep venous thrombosis (Class IIa, Level of Evidence C) [4]. Similarly, a clear reduction in the event rate by surgical or percutaneous closure of PFO as compared to medical treatment has not been fully demonstrated [5, 6]. Insufficient data exist to make a recommendation about PFO closure following a first ischemic stroke. PFO closure may be considered for patients with recurrent cryptogenic stroke despite optimal medical therapy (Class IIb, Level of Evidence C).

However, pending the results of randomized controlled trails [7, 8], patients with cryptogenic strokes and evidence of a PFO are variably managed based on the experience of the single centre, the availability to perform timely and comprehensive ultrasound patient assessment, and the accessibility of experienced interventional cardiologists. Discrepancies in published data can be also explained by different patient selection criteria, accuracy in stroke diagnosis, inclusion of patients older than 55 years [9–12], and *time elapsed* between the onset of stroke symptoms and PFO detection. To our knowledge, there are few studies on PFO detection and treatment in patients evaluated in the acute phase of an ischemic stroke or shortly after a TIA, when the risk of stroke recurrence is high [13, 14].

Aims of this study were (i) to investigate clinical and outcome measures in patients with and without a PFO among patients with undetermined stroke etiology, (ii) to compare the rate of recurrent cerebrovascular events in the short-term (i.e., at 1 week, 1 month, and 3 months) and long-term (i.e., at two years) period between medically treated PFO and PFO-negative patients, and (iii) to assess the rate of recurrent adverse events in a small subgroups of patients who *were treated with* percutaneous PFO closure.

## 2. Materials and Methods

*Patients were selected from consecutive patients admitted to our stroke unit within 24 hours from symptom onset.* Demographics and clinical data were collected. Stroke severity was assessed by the NIH Stroke Scale (NIHSS) and the modified Rankin Score (mRS). Routine diagnostic evaluation included telemetry, ultrasound echocolor Doppler study of the intracranial and extracranial vessels, and two-dimensional transthoracic echocardiography (TTE). Brain MRI with diffusion-weighted (DW) and perfusion-weighted (PW) images and MR angiogram (MRA) of the intracranial vessels were performed, if not contraindicated. Ancillary tests as screening for prothrombotic abnormalities (protein C deficiency, protein S deficiency, anti-thrombin III deficiency, factor V Leiden, and prothrombin 20210GA mutation, anti-cardiolipin IgM and IgG antibodies), autoimmunity, or collagenopathy were performed if clinically indicated. Stroke etiology was based on the Trial of Org 10172 in Acute Stroke Treatment, or TOAST criteria [15] on completion of diagnostic tests.

Eligible patients were those with no history of classic vascular risk factors (“pure” cryptogenic) as well as patients with the following MRI characteristics: (i) single subcortical lesion (<15 mm lesion) despite well-controlled vascular risk factors for small vessel disease (hypertension, diabetes), (ii) large (≥15 mm lesion) or scattered ischemic lesions in 1 vascular territory, and (iii) multiple ischemic lesions in multiple vascular territories [16, 17]. Patients in groups (ii) and (iii) had no evidence of artery-to-artery embolism (high-grade large artery stenosis, ulcerated or complicated plaque, or arterial dissection) or cardioembolic sources (atrial fibrillation, intracardiac thrombus, endocarditis, hypokinetic left ventricle wall, or ejection fraction inferior to 30%) despite coexisting classic vascular risk factors not explaining the clinical syndrome.

The contrast-enhanced Transcranial Doppler ultrasound (c-TCD) and contrast-enhanced Trans-Esophageal Ecocardiography (c-TEE) protocols we have adopted to detect PFO have been previously described [18]. Briefly, c-TCD monitoring for embolic signals passing through the middle cerebral artery (MCA) was performed using a Trans-Cranial and Vascular DWL Doppler System (Compumedics Germany GmbH). According to a standardized examination procedure [19], c-TCD was performed at rest and after Valsalva manoeuvre, using an 18-gauge needle inserted into the cubital vein with the patient in the supine position. The contrast agent was prepared using 9 mL isotonic saline solution, 1 mL of air, and 1 mL autologous blood agitated by two syringes attached via a 3-way stopcock and injected as a bolus. All studies were analyzed by two observers (FS, SDL). Severity of the RLS was defined based on the number of passing microbubbles (MB) in four categories: (i) absent (zero MB), (ii) mild (1 to 10 MB), (iii) moderate (>10 MB without curtain), and (iv) severe (“curtain” or “shower”).

All c-TEE studies were performed using a Philips Ultrasound System HD11 (Bothell, WA USA) by two cardiologists (E. D. Marchis, M. Borzi) blinded to the results of the c-TCD. During the exam the patients were alert or under mild sedation (midazolam 0.1 mg/kg). Local anaesthesia of the pharynx was obtained in all patients by xylocaine spray 0.1%. An atrial septal aneurysm (ASA) was defined as ≥11 mm of phasic septal excursion into either atrium [20, 21]. Severity and direction of the interatrial shunts were defined by color Doppler at rest and after Valsalva manoeuvre, before and after injection of agitated contrast saline solution (9 mL of isotonic saline, 1 mL of air, and 1 mL autologous blood) rapidly injected into the antecubital vein by two syringes attached via a 3-way stopcock. PFO was determined to be present if on saline contrast injection there was appearance of at least 1 microbubble in left atrium within 3 cardiac cycles after opacification of right atrium. The shunt was defined mild (<10 microbubbles), moderate (>10 microbubbles), and severe (marked opacification of the left atrium). PFOs with either >2 mm separation of septum secundum and primum or >10 microbubbles appearing in the left atrium were classified as large. All other PFOs were classified as small. Patients who were not collaborating or had lack of compliance to TEE underwent to contrast-enhanced Trans-Thoracic Echocardiography (c-TTE) by Philips Ultrasound System HD11 (Bothell, WA USA) from an experienced cardiologist (E. D. Marchis) blinded to the results of the c-TCD. Detection and grading of *right-to-left shunt* RLS was based on the same criteria adopted for c-TEE. 

### 2.1. Secondary Stroke Prevention and Clinical Follow-Up

Treatment decision (medical therapy or percutaneous PFO closure) was based on the current guidelines and patient preference. Prescribed antiplatelets were daily acetylsalicylic acid 100 mg to 300 mg or clopidogrel 75 mg. Oral anticoagulants were prescribed to patients with recurrent strokes despite antiplatelets, coexisting ASA, prothrombotic conditions, or deep venous thrombosis (the target international normalized ratio was between 2 and 3). Selected cases were referred to experienced interventional cardiologists for percutaneous closure of PFO. Follow-up *clinical *evaluations were performed at 1, 3, 6, and 24 months after the index event. At each time-point information on neurological and functional status, adverse events, compliance to the prescribed medications, life-style changes (physical activity, changes in diet and weight, and cigarette smoking cessation), and information on vascular risk factor control were collected. A recurrent ischemic stroke was considered in the presence of acute onset of focal neurological signs of more than 24 hours' duration with evidence of a new ischemic lesion on CT or MRI scan, or when new lesions were absent but clinical syndrome was consistent with stroke. A recurrent TIA was considered in the presence of acute onset of focal neurological signs of less than 24 hours' duration with or without evidence of a new ischemic lesion on DW-MRI scan. 

For statistical analyses, we compared clinical, imaging, and outcome measures between patients with and those without a PFO using Student's *t*-test (for quantitative variables), and contingency tables with Fisher's test (for categorical variables). The rates of stroke recurrence or death in the short period (i.e., at 1 week, 1 month, 3 months) and in the long term (i.e., at two years) after the index event were assessed. Factors independently associated with favourable outcome (mRS ≤ 2) at 12 months were identified by logistic regression analysis. ORs with 95% CIs were calculated. *P* < 0.05 was considered statistically significant. The analysis was performed by the Statistica 7 software. 

## 3. Results

Over 18 months (January 2007 to June 2008) 674 patients with a diagnosis of acute ischemic stroke or transient ischemic attack (TIA) were admitted in our stroke unit. There were 148/674 (22%) patients with uncertain stroke etiology. Of them, 19/148 (13%) of patients were excluded from the analysis for contraindications to MRI study (*n* = 10; 63%), lack of compliance to c-TEE or c-TTE (*n* = 5; 26%), and dropout at follow-up evaluation (*n* = 4; 21%), leaving 129/148 (87%) eligible for the study. A PFO was detected in 57/129 (44%) patients. Compared to PFO-negative patients, those with a PFO were more frequently females (48% versus 34%; *P* = 0.02), had similar vascular risk factors except for higher occurrence of hypercholesterolemia (30% versus 16%; *P* = 0.03), and had lower NIHSS score at 24 hours (3 versus 5; *P* = 0.04). A *statistically nonsignificant* tendency was observed for higher occurrence of stroke onset on awakening in PFO patients (*P* = 0.09) (These results are shown in Table 1). At logistic regression analysis (with age, sex, stroke risk factors, presence of PFO, stroke severity, type, and location of infarct as independent variables) NIHSS at onset (OR 0.55; 95% CI, 0.31 to 0.80; *P* < 0.001) and NIHSS at 24 hours (OR 0.70; 95% CI, 0.49 to 0.91; *P* < 0.001) were negatively associated with favourable outcome after two years (mRS ≤ 2).

Patients with a PFO were treated for secondary stroke prevention based on the current guiding principle, also taking into account the following aspects: patients' age and favourite therapeutic approach; previous history of TIA or stroke and the presence of chronic ischemic lesions on MRI scan, presence of vascular risk factors and if they were well controlled before the index stroke or TIA, and degree of the *right-to-left shunt* (RLS) on c-TCD and its correlation with the morphological characteristics and amplitude of PFO on c-TEE. Prescribed antithrombotic treatment was acetylsalicylic acid 100 to 300 mg o.d. (*n* = 23), clopidogrel 75 mg o.d. (*n* = 18), and warfarin 5 mg o.d. (*n* = 6). Patients were started on warfarin if they had prothrombotic conditions, history of deep venous thrombosis, coexisting ASA, a lacunar infarct or infarct involving less than 1/3 on the MCA territory, and if the risk of bleeding was deemed acceptable based on age, cognitive status, comorbidities, presence of a caregiver, and compliance to treatment. Nine patients (15.7%) were referred to experienced interventional cardiologists for catheter PFO closure: all were younger than 55 years, had moderate to severe shunt on ultrasound studies, and all preferred the percutaneous PFO closure *instead medical treatment*. *Treatment with acetylsalicylic acid 100 mg o.d. was started after the procedure*. The interventional procedure was uneventful in all patients and no residual shunt was observed in none of the treated patients.

There were no differences between patients with and those without a PFO at two years in motor and functional outcome measure (Table 2). The rates of recurrent stroke and TIA did not significantly differ between patients with and without a PFO: 0.0% versus 1.4% at 1 week, 1.7% versus 2.7% at 1 month, and 3.5% versus 4.2% at 3 months, respectively. The two-year rate of recurrent stroke or TIA was 5/48 (10.4%) in medically treated PFO patients and 6/72 (8.3%) in PFO-negative patients (*P* = 0.65). In PFO-positive patients the recurrent event occurred at 1, 4, 15 (*n* = 2), and 23 months after the index event; alternative causes for recurrent stroke were detected in all patients: an ulcerated aortic plaque was detected in 3 patients, while atrial fibrillation was diagnosed in 2 patients; of note, atrial fibrillation was an incidental finding during the follow-up in 2 other patients. In PFO-negative patients the recurrent event occurred at 7 days, at 3, 9, 18, and 21 months after the index event; 2 patients died at 1 and 7 months (one massive intracranial bleeding while on warfarin, and one fatal myocardial infarction). An intracardiac thrombus was detected at the time of the recurrent stroke in one patient, while a large aortic plaque was detected in another patient who had recurrent stroke. All patients *treated with percutaneous* PFO closure (*n* = 9) were alive after two years, and none of them had recurrent cerebrovascular events.

## 4. Discussion

We investigated the clinical benefit of early screening for PFO in patients with an acute ischemic stroke or TIA of undetermined etiology. The rate of early and two-year recurrent stroke or TIA in patients with a PFO under best medical treatment was compared to those of patients with no evidence of a PFO. The rates of recurrent stroke and TIA in PFO and no-PFO patients in the short-term did not significantly differ between the two groups: 0.0% versus 1.4% at 1 week, 1.7% versus 2.7% at 1 month, and 3.5% versus 4.2% at 3 months, respectively. These rates are lower than those previously observed in patients with undetermined stroke etiology: the risk of recurrent cerebrovascular events observed by Lovett et al. [14] was 2.3% at 7 days, 6.5% at 1 month, and 9.3% at 3 months, which were similar to those observed in cardioembolic strokes. However, in this study, the analysis was not restricted to patients with PFO. Ongoing randomized controlled studies should be able to provide further information on early rate of adverse events in PFO patients [7, 8]. In our study, the rate of recurrent cerebrovascular events did not significantly differ between PFO medically treated patients and no-PFO patients (10.4% and 8.3%, resp.; *P* = 0.65), where a relative risk (RR) of 1.25 for the presence of a PFO was observed. In earlier studies, the event rate (recurrent stroke or death) in patients with PFO younger than 55 years was relatively low, being about 2.0% (1.6–2.4%) per year, with a reported average recurrence rate for stroke or TIA in medically treated patients of 4.0% for the first year and 8.6% within 2 years [2, 22, 23]. In a recent meta-analysis of 15 studies [24] the pooled absolute rate of recurrent ischemic stroke or TIA at one year in patients with PFO treated medically was 4.0% (95% CI 3.0 to 5.1) while the rate of recurrent ischemic stroke was 1.6% (95% CI 1.1 to 2.1). The pooled RR for recurrent ischemic stroke or TIA in patients compared to those without a PFO was 1.1 (95% confidence interval (CI) 0.8 to 1.5). For ischemic stroke, the pooled RR was 0.8 (95% CI 0.5 to 1.3). *A low recurrence rate of stroke has been recently reported in 108 young patients (aged 18–45 years) with cryptogenic ischemic stroke with and without PFO [25]. In this long-term follow-up study (patients were followed up to 66 months) the average annual rate of recurrent cerebral ischemia was 1.1% and 1.6% for patients with and without PFO, respectively. In this study the recurrence rate did not increase with the presence of PFO, ASA, or other variables.*

Taken together, these data indicate that the risk of recurrent stroke after a cryptogenic stroke is not significantly increased in patients with PFO under medical treatment as compared to patients without a PFO. *As recently reported by single centre [26, 27], as well as by a multicenter Italian study [28], we also observed that percutaneous PFO closure may be superior to medical treatment in preventing stroke recurrence. However, there is evidence suggesting that this approach may be less effective in older patients [29].*

Differences in the rates of recurrent cerebrovascular events may be explained by great heterogeneity among studies. First, we observed a significant female prevalence among patients with a PFO compared to PFO-negative patients (48% versus 34%), which was similar to the percentages reported by Lamy et al. (48% versus 38%; *P* = 0.02) [30]. In a recent meta-analysis [24] and with the exception of one Italian study [22], a male prevalence was reported either in studies with or in those without a non-PFO comparison group. A difference in sex prevalence might be related to undetermined factors predisposing to paradoxical embolism, but this remains speculative. Second, in our study stroke severity at onset did not differ between patients with and without a PFO, strokes were of mild severity, and the 3-month outcome was good in both groups. Stroke severity in PFO patients has been rarely reported in published series: in the study of Bogousslavsky et al. [23], a low rate of stroke recurrence was contrasted with the severity of initial stroke, which was disabling in one-half the patients. Further, in their study there were fewer TIAs (16% versus 23% in our study) and an alternative cause of stroke was present in 16% of patients, usually cardiac (atrial fibrillation, severe mitral valve prolapse, akinetic left ventricular segment); conversely, in the study of Lamy et al. [30], more than half of patients with PFO had mild strokes as measured by an mRS of 0-1, *and the outcome was favourable in more than 60% of cases in the study of Arauz et al. *[25]. Third, our patients were older compared to previous reports (about 2/3 of patients were older than 55 years). The slightly increased two-year rate of recurrent cerebrovascular events observed in our study as compared to previous analyses might be attributed to the inclusion of patients with classic vascular risk factors for stroke and older than 55 years, who are at higher risk of developing factors associated with stroke recurrence in cryptogenic stroke as aortic arch plaque [31], or atrial fibrillation (which was diagnosed at stroke recurrence in two PFO patients, and it was an incidental finding at follow-up in two other PFO patients). Further, in our study, a PFO was associated with ASA in 41% of patients, which is higher than previously reported (17–24%) [20, 30, 32, 33]. However, whether ASA alone or in association with a PFO confers an increased risk of stroke recurrence in medically treated patients is still debated [2, 3, 20, 34]. Finally, although not significant, we observed that patients with a PFO had more frequently strokes-on-awakening. This has been previously reported and associated with obstructive sleep apnoea causing right atrial pressure elevation during the night resulting in RLS through a PFO [35]. We recently observed that the administration of a single oral dose of sildenafil (an inhibitor of phosphodiesterase type 5) is able to affect the interatrial pressure gradient by acting on pulmonary resistances thus reducing severity of RLS on c-TCD [36]. A change in RLS volume on c-TCD ultrasound over time has been recently observed in 1/3 of patients with cryptogenic stroke [37]. These data suggest that large multicentre observational studies are warranted in order to discriminate subgroups of PFO patients at higher risk of stroke recurrence from those with incidental PFO as well as to develop new therapeutic approaches for secondary stroke prevention based on cardiac and pulmonary hemodynamic parameters.

This study has some limitations. This was a single centre observational study, with a relatively small sample size, and our results cannot be generalized. Further, we did not systematically investigate those age-related factors thought to increase the risk of paradoxical embolism (i.e., pulmonary artery pressure, prothrombotic states, deep venous thrombosis) as well as their change over time. In addition, we did not test patients for persisting RLS at follow-up examination [37].

In conclusion, while pending evidence-based guidelines for PFO management [7, 8], all available treatment options and drawbacks keep to be explained and offered to the patient, and the patient's preference and fear should be taken into account [38]. In this setting, single-centre studies performed in highly selected stroke populations may guide personalized prevention treatment and test adjuvant treatment options based on the increasing insight into the pathophysiology of stroke in patients with PFO.

## Figures and Tables

**Table 1:** Differences in demographic, clinical, and vascular risk factors between patients with a PFO confirmed by combined *ultrasound* approach and those with negative TCD study for RLS (i.e., PFO-negative patients).

	PFO (+) patients *n* = 57	PFO (−) patients *n* = 72	*P *value
Age, mean (±SD) y.o.	57 (±14)	60 (±14)	0.57
Age ≤55 y.o., *n* (%)	21 (37%)	26 (36%)	0.67
Female sex, *n* (%)	27 (48%)	25 (34%)	0.02
Vascular risk factors, *n* (%):			
Hypertension*	28 (49%)	32 (50%)	0.59
Diabetes mellitus*	8 (14%)	12 (17%)	0.91
Hypercholesterolemia*	17 (30%)	12 (16%)	0.03
Smoking^§^	21 (37%)	35 (48%)	0.12
On antiplatelets before stroke/TIA, *n* (%)	14 (25%)	19 (26%)	0.86
Previous TIA or stroke, *n* (%)	11 (19%)	12 (17%)	0.52
On statins before stroke/TIA, *n* (%)	7 (12%)	5 (7%)	0.09
Previous ischemic lesions on MRI, *n* (%)	39 (68%)	55 (76%)	0.25

*Known before stroke or TIA.

^§^Current or past (less than 5 years) smoking.

**Table 2:** Differences in clinical and radiological characteristics of the index event and two-year outcome between patients with a PFO confirmed by combined *ultrasound* approach and those with negative c-TCD study for RLS (i.e., PFO-negative patients).

	PFO (+) patients *n* = 57	PFO (−) patients *n* = 72	*P* value
Index event: TIA/stroke, *n* (%)	10 (17.5%)	20 (27%)	0.1
Single/multiple/no acute lesion, *n*	37/10/10	35/17/20	0.1
Anterior circulation stroke (MCA), *n* (%)	32 (56%)	40 (55%)	0.78
Symptoms on awakening, *n* (%)	10 (17.5%)	8 (11%)	0.09
Onset NIHSS score, mean (±SD)	5 (±4)	6 (±5)	0.3
24-hour NIHSS score, mean (±SD)	3 (±3)	5 (±4)	0.04
Discharge NIHSS score, mean (±SD)	2 (±3)	3 (±3)	0.11
Days of in-hospital stay, mean (±SD)	4.4 (±2.1)	4.5 (±2.7)	0.3
Three-month outcome			
NIHSS score, mean (±SD)	2 (±2)	2 (±2)	0.28
mRS score, mean (±SD)	2 (±1)	1 (±2)	0.35
BI score, mean (±SD)	92 (±24)	97 (±25)	0.19
Two-year rate of stroke recurrence or death	5/48 (10.4%)*	6/72 (8.3%)	0.65

MCA indicates middle cerebral artery; NIHSS: National Institute of Health Stroke Scale; mRS: modified Rankin Scale; BI: Barthel Index. *9/57 (15.8%) patients underwent to percutaneous PFO closure and no recurrent events or death were recorded in this group.
